# Identification of Metabolic risk phenotypes predisposing to Non-Alcoholic Fatty Liver Disease in a Pakistani Cohort

**DOI:** 10.12669/pjms.331.11445

**Published:** 2017

**Authors:** Rizwana Abdul Ghani, Muhammad Saqlain, Muhammad Mobeen Zafar, Shagufta Jabeen, Syed Muhammad Saqlan Naqvi, Ghazala Kaukab Raja

**Affiliations:** 1 Rizwana Abdul Ghani, PhD. Department of Biochemistry, Pir Mehr Ali Shah Arid Agriculture University Rawalpindi, Murree Road, Rawalpindi, Pakistan; 2 Muhammad Saqlain, M.Phil. Department of Biochemistry, Pir Mehr Ali Shah Arid Agriculture University Rawalpindi, Murree Road, Rawalpindi, Pakistan; 3 Muhammad Mobeen Zafar, M.Phil. Department of Biochemistry, Pir Mehr Ali Shah Arid Agriculture University Rawalpindi, Murree Road, Rawalpindi, Pakistan; 4 Shagufta Jabeen, PhD. Department of Biochemistry, Pir Mehr Ali Shah Arid Agriculture University Rawalpindi, Murree Road, Rawalpindi, Pakistan; 5 Syed Muhammad Saqlan Naqvi, PhD. Department of Biochemistry, Pir Mehr Ali Shah Arid Agriculture University Rawalpindi, Murree Road, Rawalpindi, Pakistan; 6 Ghazala Kaukab Raja, PhD. Department of Biochemistry, Pir Mehr Ali Shah Arid Agriculture University Rawalpindi, Murree Road, Rawalpindi, Pakistan

**Keywords:** Obesity, Hyperglycemia, Dysglycemia, Hypertension, NAFLD

## Abstract

**Objectives:**

Non-alcoholic fatty liver disease (NAFLD) has emerged in the last two decades with worldwide prevalence of 25.24%. Due to its increasing frequency in Pakistan, it was aimed to identify disease predisposing metabolic risks and their association with NAFLD.

**Methods:**

Anthropometric and biochemical investigations were collected from 1366 subjects with minor metabolic disturbances. Comparative analyses were performed to compute frequencies of common metabolic risk phenotypes while their associations with NAFLD were explored using regression analyses. The prevalence of NAFLD was also estimated in total, age, and gender-based population cohorts.

**Results:**

Among metabolic risk phenotypes obesity, hyperglycemia, hypertension, and dyslipidemia significantly associated (*p*<0.001) with NAFLD risk irrespective of age, gender, and BMI. Prevalence of NAFLD in total study cohort was 14.8%, 16.1% in males, 13.4% in females, 19.9% in ≥40 years and 8.7% in ≤40 years respectively.

**Conclusion:**

General Pakistani populations experiencing common metabolic disturbances are at high risk of NAFLD development, especially male gender and advanced age. Based on these parameters the stratified NAFLD population could be treated accordingly.

## INTRODUCTION

Non alcoholic Fatty liver disease or NAFLD is said to result from a spectrum of disorders like insulin resistance, diabetes mellitus and obesity possibly due to unhealthy and sedentary life style.^1^-^3^ Generally NAFLD is not considered as a serious illness, if remains un-diagnosed or untreated could progress to advanced hepatic complications and liver cirrhosis. Though it was the major health issue of western countries.^4^ NAFLD is now given full consideration in Asia especially due to the rise in over-weight/obesity and related metabolic health complications.^5^,^6^ The global prevalence rate of NAFLD is reported to be 25.24% while in Asian countries it ranges between 15–45%.^4^,^7^ As per World Gastroenterology Organization Global Guidelines 2014, the NAFLD prevalence rate in general Pakistani population is 18%.^3^ A number of small population based local studies have reported general as well as gender specific NAFLD frequency rates and disease risk factors.^8^,^9^ As globally declared, the alarmingly increasing frequency of NAFLD in Pakistan could be a manifestation of gradual rise in over-weight/obesity along with common metabolic health complications.^4^-^7^,^10^,^11^

Keeping in mind the unhealthy lifestyle transformations leading to obesity and common metabolic dysregulations particularly in the urban Pakistani populations, the major question addressed in present study was to explore the susceptibility of Pakistani populations towards NAFLD development. The major focus of study was to explore frequencies of NAFLD and common metabolic risk phenotypes as well as associations among them.

## METHODS

A total of 1366 subjects visiting out-patient departments (OPDs) of public hospitals from Rawalpindi and Islamabad, Pakistan were included in this study keeping with the inclusion exclusion criteria. The study was approved from the Ethics Committee for the use of Human Subjects, PMAS-AAUR. Informed consent was obtained from subjects and data was collected using questionnaire. Anthropometric data (age, sex, weight, height, blood pressure) was collected. The clinical assays values for Fasting Blood Sugar (FBS), Total triglycerides (TG), Total Cholesterol (TC), High Density Lipoprotein Cholesterol (HDL-C), Low Density Lipoprotein Cholesterol (LDL-C) and liver enzymes (Alanine Aminotransferase (ALT), Aspartate aminotransferases (AST), Alkaline Phosphatase (ALP) were obtained. The inclusion criterion was based on having at least 2-3 of the following metabolic risk phenotypes; overweight/obesity, hyperglycaemia, dyslipidemia, hypertension with age ranging from 20-65 years. The subjects with pregnancy, viral hepatitis, advanced stage liver disorder(s), being on lipid lowering drugs or using alcohol were not included. Based on the elevated ranges of anthropometric parameters and biochemical assays profiles; Obesity (BMI>29.9Kgm^2^), hyperglycemia (FBS≥100mg/dL), dyslipidemia (TG≥150mg/dL and HDL<40mg/dL for men and <50mg/dL for women) and hypertension (SBP≥130mmHg/DBP≥85mmHg) subjects being at risk of NAFLD were identified. While NAFLD status of study subjects with elevated ALT levels (>55 IU/l in men and >33 IU/l in females) along with disturbed levels of other biochemical assays profiles and anthropometry was confirmed by the physician/gastroenterologists from respective hospitals.

The data analysis was carried out using SPSS version 16.0 software (SPSS Inc., Chicago, IL, USA). The descriptive statistics (Means and SD) were used to summarize the collected data in all study subjects as well as in those with elevated levels of risk factors and in gender and age based groups (≤40years and ≥40years) using one way analysis of variance (ANOVA). Based on anthropometric data and biochemical tests values, prevalence of NAFLD in total, gender and age based groups was estimated. Comparison of NAFLD frequencies and confirmation of statistically significant differences in gender, age, and NAFLD vs normal population groups with unequal number of subjects was done using proportion difference test. Based on elevated levels of clinical markers, the frequencies of four major risk phenotypes, viz., Obesity (BMI>29.9Kgm^2^), hyperglycemia (FBS≥100mg/dL), dyslipidemia (TG≥150mg/dL and HDL<40mg/dL for men and <50mg/dL for women) and hypertension (SBP≥130mmHg/DBP≥85mmHg) were also estimated in total and NAFLD risk populations. The associations of metabolic risk phenotypes with NAFLD were performed using binary and multinomial logistic regression (OR at CI 95%) in unadjusted as well as gender, age, and BMI adjusted data. A significance level of ≤0.05 was used in all statistical analyses.

## RESULTS

The total disease prevalence rate in our study cohort was 14.8% (Table-I). The gender specific disease prevalence rate was 16.1% in male subjects as compared to 13.4% in females. In age based population groups, the ≥40 years group had significantly higher NAFLD prevalence rate of 19.9% (*p*<0.001) as compared to 8.7% in ≤40 years (Table-II).

**Table-I:** Comparison of Disease Risk Factors and NAFLD Prevalence in Total Study Cohort.

Variables	Total Subjects (MEAN±SD)	NAFLD Subjects (MEAN±SD)
Age (years)	41.6±9.7	45.7±8.1
BMI (Kg/m^2^)	24.4±4.8	28.6 ±6.26
SBP (mmHg)	125.8±14.9	134.7 ±17.8
DBP (mmHg)	84.7±11.4	92.1 ±12.46
BSF (mg/dL)	102.1±46.8	135.0 ±71
TG (mg/dL)	142.4±83.4	198.0 ±110
TC (mg/dL)	172.7±49.4	200.0 ±51.8
HDL (mg/dL)	45.7±12.8	37.9 ±11.3
LDL (mg/dL)	105.1±33.7	120.0 ±45
ALT (U/L)	29.1±14.5	49.17 ±16.19

Total Disease Prevalence 14.8%

**Table-II:** Gender and Age Based Comparison of Disease Risk factors and NAFLD Prevalence.

Variables	Gender Based Disease Status		p-value	Age Based Disease Status		p-value

	Males (MEAN±SD)	Female (MEAN±SD)		<40 years (MEAN±SD)	>40 years (MEAN±SD)
Age (years)	42.3±9.8	41.06±9.7	0.2	32.44±5.06	49.5±4.7	<0.001
BMI (Kg/m^2^)	27.7 ±6.04	30.24 ±6.2	0.002	27.53 ±5.8	29.1 ±6.3	0.117
SBP (mmHg)	136.06 ±18.02	135 ±17.6	0.25	133 ±16.0	135 ±18.0	0.49
DBP (mmHg)	93.23 ±12.2	92.9 ±12.7	0.177	91 ±10.65	92 ±11.57	0.588
BSF (mg/dL)	130.9 ±20.5	144.5 ±32	0.123	121.36 ±34.7	140.45 ±53.7	0.096
TG (mg/dL)	203.4±52.4	195.20±50.8	0.264	209.88 ±52.1	193.2 ±52.4	0.764
TC (mg/dL)	207.48 ±12.4	185.9 ±107.8	0.169	197.88 ±52.1	200.2 ±52.4	0.39
HDL (mg/dL)	37.52 ±11.8	38.4 ±10.8	0.586	38.11 ±10.8	37.84 ±11.6	0.881
LDL (mg/dL)	120.28±50	120.56±40.1	0.966	115.11 ±38	122.34 ±48.4	0.232
ALT (U/L)	48.72 ±15.6	49.79 ±16.9	0.656	49.56 ±15.7	49.03 ±16.4	0.838
Disease Prevalence (%)	16.1	13.4	0.077	8.7	19.9	<0.001

The mean values of all studied parameters were raised while HDL lower than the normal ranges in NAFLD/high risk subjects (*p*<0.001) as compared to the total population (Table-I). The gender and age based disease groups lacked significant difference with all risk markers except BMI which was significantly elevated in female study subjects (30.24±6.2, *p*=0.002).

**Fig. 1:**
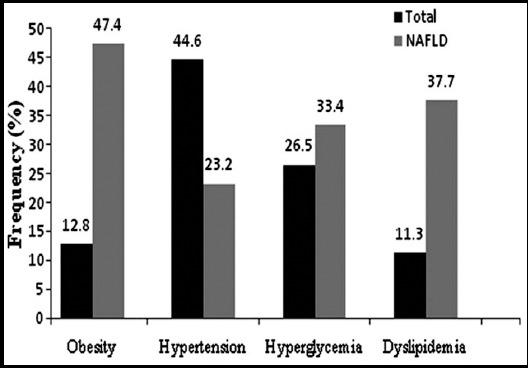
Frequencies of Metabolic Risk Phenotypes in Total Population Cohort and NAFLD Subgroup.

The frequencies of all risk phenotypes are presented in Figure 1. Except hypertension, obesity, hyperglycemia, and dyslipidemia all were raised in NAFLD group as compared to the total study cohort. All metabolic risk phenotypes significantly associated with NAFLD in both unadjusted and adjusted models (Table-III). In unadjusted model, except gender, all metabolic phenotypes were significantly associated with NAFLD in ≥40 years, raising 2.6 folds higher risk of disease (OR=2.60, *p*=1x10^-6^) in older subjects as compared to the younger age group (<40 years). In age, gender and BMI adjusted model, the strongest association with NAFLD was with obese state which increased 4.2 folds higher risk of disease (OR=4.2) followed by hyperglycemia (OR=3.5), hypertension (OR=1.95), and dyslipidemia (OR=1.6). The gender lacked association with disease in un-adjusted model (*p*=0.29) but after adjustment, the risk of NAFLD development increased to 1.74 folds in male subjects (OR=1.74, *p*=0.002) as compared to females. Likewise, data adjustment significantly raised the risk of NAFLD (OR=1.8, *p*=0.001) in advanced age (≥40.0years) as compared to <40 years.

**Table-III:** Association Analysis of Metabolic Risk Phenotypes with NAFLD.

Metabolic Risk Phenotypes	Un-adjusted Odds Ratio (95%CI)	p-value	Age, Gender, BMI-Adjusted Odds Ratio (95%CI)	p-value
Gender (Female vs Male)	1.2 (0.92-1.7)	0.29	1.74 (1.2-2.5)	0.002
Age (<40 vs >40 Years)	2.60 (1.86-3.63)	<0.001	1.8 (1.3-2.6)	0.001
Hyperglycemia (FBS>100mg/dL)	5.3 (3.80-7.35)	<0.001	3.5 (2.5-5.0)	<0.001
Obesity (BMI >29.9Kg/m^2^)	4.8 (3.45-6.67)	<0.001	4.2 (2.7-6.3)	<0.001
Dyslipidemia (TG>200mg/dL & HDL<40mg/dL)	4.1 (2.80 5.95)	<0.001	1.6 (1.04-2.5)	0.03
Hypertension (SBP≥130mmHg/DBP≥85mmHg)	3.1 (2.21-4.26)	<0.001	1.95 (1.4-2.8)	<0.001

## DISCUSSION

In present study we report major metabolic risk phenotypes; obesity, hyperglycemia, hypertension, and dyslipidemia being the strongest contributors of NAFLD in general Pakistani populations. Our results further highlight higher disease susceptibility in male subjects and advanced age (>40 years) when already exposed to common metabolic disturbances. The prevalence of NAFLD found among total study cohort was 14.8% which increased to 16.1% in male subjects and to further 19.9% among >40 years old subjects. Thus clustering of common metabolic risk phenotypes in general Pakistani populations increase the risk of fatty liver development many folds.

The metabolic risk phenotypes; obesity, hyperglycemia, dyslipidemia, and hypertension have been globally declared as major health risks increasing rates of world populations suffering from hepatic steatosis regardless of age and gender.^7^ Global prevalence of NAFLD is 25.24% (95% CI: 22.10-28.65) with highest prevalence in the Middle East and South America and lowest in Africa. The internationally reported 18% NAFLD prevalence rate for general Pakistani population while present study reports 14.8%. A study by Niaz *et al*. reported NAFLD frequency rates of 13.5% in healthy and young male subjects.^9^ The relatively lower disease prevalence rate could be reflective of population cohort selection criterion in our study; over-weight/obese subjects experiencing hyperglycemia, dyslipidemia, or hypertension. It could be suggestive that the majority of study population with metabolic disturbances might not yet be exposed to fatty liver development stage or might have lower susceptibility towards NAFLD. The gender based NAFLD prevalence reported from this study is somewhat contradictory, compared to another study reporting females being at higher disease risk as compared to males.^11^ Our study population lacked gender specific disease prevalence rates thus showing males and females with metabolic risks clustering are equally susceptible to NAFLD. Likewise, the unadjusted disease association analyses with gender also lacked statistical significance (Table-III), however the age, gender and BMI adjusted model improved disease association, increased 1.2 odds higher risk of developing NAFLD in males as compared to females in our study cohort (Table-III). A number of recent studies have reported higher NAFLD prevalence rates in males and male gender as a risk factor for fatty liver disease.^12^,^13^ As for the physical factors, generally advanced age is considered as a strong predictor of obesity, T2D, CVDs, and other metabolic complications, however its role in NAFLD is disputable.^9^ In our study cohort, age proved to be a strong predictor of disease risk, >40 years age group was at significantly higher odds of developing NAFLD thus suggesting that this age group might be more prone to accumulation of hepatic fats along with other metabolic complications. A study by Goh *et al*. on multiracial Asian populations residing in Malaysia also reported higher risk of NAFLD in male gender and older age group when compared to female gender and younger age subjects.^14^ In a study on diabetic Pakistani patients, male subjects were found at higher NAFLD risk as compared to females.^15^ Likewise younger, healthhy Pakistani male subjects were reported with 13.5% disease risk.^9^ Therefore it could be worth to mention that the overall health status of an individual might be the major contributor of excess fat depots in liver regardless of age and gender.

Being major focus of this study, the frequencies of obesity, hyperglycemia, dyslipidemia, and hypertension, being potential NAFLD predisposing markers, were estimated in total cohort and in NAFLD sub-group (Fig.1). The associations of all four risk phenotypes with NAFLD computed in both unadjusted and age, gender and BMI adjusted models (Table-III) provide strong evidence that clustering of common metabolic disturbances in general Pakistani populations could be considered as a major threat of increasing frequency of NAFLD. Though in an unadjusted population data hyperglycemia was strongly associated with disease risk, obesity proved to be strongest disease predictor of NAFLD after data adjustments. The subjects already diagnosed with T2D are found to be more susceptible to NAFLD as compared to non-diabetic irrespective of gender.^8^,^15^ Obesity has already been declared globally to over-burden major metabolic pathways and these dysregulations could affect two thirds of the obese subjects as compared to one third of the total adult world population.^1^,^7^ In Pakistan general as well as central/abdominal obesity has increased at an alarmingly high rate.^16^ Over-eating, high fat and low fiber diets along with a sedentary and stressful life style of a common person are a few obesity pre-disposing risks.^2^ Besides ethnicity predisposition towards obesity is another crucial health risk factor while exploring NAFLD among obese people.^4^-^6^ Pakistan has a rich culture with multiple ethnic and casts/sub-casts of South Asian ancestry reported for a high genetic predisposition towards weight gain and related complications.^4^,^6^ Though of prime importance, it was beyond our current study design to include vast ethnicity based information for disease prevalence and association. However ethnic predisposition towards obesity could provide useful information in clearly explaining the role of BMI as a risk marker of NAFLD.^17^ Though BMI covers overall obesity, waist circumference (WC) is considered an important surrogate marker of abdominal adiposity and a risk factor for cardiovascular and metabolic diseases including fatty liver.^18^ Due to the lack of data for whole studied cohort, WC was not included in present study analysis.

Along with obesity, hyperglycemia/altered glucose metabolism is another major health complication associated with NAFLD.^12^ Large variations (10-75%) exists in the frequency of NAFLD among type 2 diabetic subjects worldwide, thus indicating an independent correlation among these two metabolic complications.^19^,^20^ Present study data also reports a very high frequency of hyperglycemia (26.50%) significantly strong association with NAFLD (OR=5.3, p=1x10-5). Though hyperglycemia increases 5.3 folds the risk of NAFLD in an unadjusted study cohort the risk still remains 3.5 folds high after age, gender and BMI adjustments (OR=3.5, p=1x10-6). Thus increased prevalence of hyperglycemia and its association with NAFLD is an indicator of its strong role as a predictive risk marker in our population. The elevated circulating glucose levels could lead to increased insulin resistance at hepatic and peripheral levels thus contributing towards pathogenesis of NAFLD and incident T2D.(20) However after age, gender and BMI based adjustments of total study cohort, obesity takes the lead in being the strongest predictor of NAFLD (OR=4.2, p=1x10-6) followed by hyperglycemia (OR=3.5, p=1x10-5).

Another important risk parameter of metabolic complications is dyslipidemia and included among strong risk marker for NAFLD and associated complications.^21^ High plasma triglycerides and low HDL-cholesterol are major determinants of metabolic disturbances and hepatotoxicity, posing increased risks for T2D, CVDs as well as NAFLD.^22^ The overall frequency of dyslipidemia among total population of present study was 44.60%. In accordance with previous reports, subjects with dyslipidemia were at significantly higher odds of NAFLD risk in unadjusted (OR=4.1, p=1x10-5) and adjusted (OR=1.6, p=0.03) disease models as compared to those with normal lipid profile.^6^ The fourth metabolic risk phenotype recorded with high frequency in our study cohort was hypertension (Fig.1). As for disease association, the hypertensive subjects were at 3.1 and 1.95 folds higher risk of NAFLD in un-adjusted and adjusted disease models respectively (Table-III). An independent association of hypertension with NAFLD has been documented and related with increased body weight and insulin resistance while association of NAFLD with hypertension and high-normal SBP has been reported.^23^-^25^

Overall we report high prevalence of NAFLD along with obesity, hyperglycemia, dyslipidemia and hypertension in our local studied cohort. The significantly strong associations of major metabolic risk phenotypes with NAFLD especially obesity clearly indicates high susceptibility rate of NAFLD in over-weight/obese subjects already carrying the burden of common metabolic complications. Though this study cohort is not a true representative of all major ethnic groups of Pakistan and could be deficient of some important physical and biochemical disease predictors, it has highlighted the major risk factors pre-disposing general Pakistani populations to NAFLD. The findings of present study could be replicated in a bigger multi-racial study cohort to identify ethnicity specific disease marker for timely diagnosis, management, and treatment of disease.

## References

[1] Hannah WN, Harrison SA (2016). Lifestyle and Dietary Interventions in the Management of Nonalcoholic Fatty Liver Disease. Dig Dis Sci.

[2] Veena J, Muragundla A, Sidgiddi S, Subramaniam S (2014). Non-alcoholic fatty liver disease: need for a balanced nutritional source. Br J Nutr.

[3] LaBrecque DR, Abbas Z, Anania F, Ferenci P, Khan AG, Goh K-L (2014). World Gastroenterology Organisation global guidelines: Nonalcoholic fatty liver disease and nonalcoholic steatohepatitis. J Clin Gastroenterol.

[4] Farrell GC, Wong VW-S, Chitturi S (2013). NAFLD in Asia—as common and important as in the West. Nature Reviews Gastroenterol Hepatol.

[5] Ashtari S, Pourhoseingholi MA, Zali MR (2015). Non-alcohol fatty liver disease in Asia: Prevention and planning. World J Hepatol.

[6] Misra A, Shrivastava U (2013). Obesity and dyslipidemia in South Asians. Nutrients.

[7] Younossi ZM, Koenig AB, Abdelatif D, Fazel Y, Henry L, Wymer M (2016). Global Epidemiology of Non-Alcoholic Fatty Liver Disease-Meta-Analytic Assessment of Prevalence, Incidence and Outcomes. Hepatology.

[8] Afzal MY, Anjum HS, Siddiqui UN, Shahid S (2016). Nonalcoholic fatty liver disease (NAFLD) frequency in Diabetes Mellitus (DM) type–II patients. FUUAST J Biol.

[9] Niaz A, Ali Z, Nayyar S, Fatima N (2011). Prevalence of NAFLD in Healthy and Young Male Individuals. ISRN Gastroenterology.

[10] Ullah I, Qureshi MS, Hadi A, Khan SB, Hafizullah M (2014). Correlation of body mass index with frequency of high blood pressure. Pak Heart J.

[11] Ahmed S, Ahmed SA, Ali N (2010). Frequency of metabolic syndrome in type 2 diabetes and its relationship with insulin resistance. J Ayub Med Coll Abbottabad.

[12] Shams ME, Al-Gayyar MM, Barakat E (2011). Type 2 Diabetes Mellitus-lnduced Hyperglycemia in Patients with NAFLD and Normal LFTs: Relationship to Lipid Profile, Oxidative Stress and Pro-Inflammatory Cytokines. Sci Pharm.

[13] Zhou Y, Llauradó G, Orešič M, Hyötyläinen T, Orho-Melander M, Yki-Järvinen H (2015). Circulating triacylglycerol signatures and insulin sensitivity in NAFLD associated with the E167K variant in TM6SF2. J Hepatol.

[14] Goh S-C, Ho EL-M, Goh K-L (2013). Prevalence and risk factors of non-alcoholic fatty liver disease in a multiracial suburban Asian population in Malaysia. Hepatol Int.

[15] Seetlani NK, Memon AR, Tanveer S, Ali A, Ali P, Imran K (2016). Frequency of Non-Alcoholic Steatohepatitis on Histopathology in Patients of Type 2 Diabetes Mellitus with Duration of More than 5 Years. J Coll Physicians Surg Pak.

[16] Mahmood M, Ashraf T, Memon MA, Achakzai J (2010). Abdominal obesity pattern among various ethnic groups presenting with acute coronary syndrome. J Ayub Med Coll Abbottabad.

[17] Bedogni G, Miglioli L, Masutti F, Tiribelli C, Marchesini G, Bellentani S (2005). Prevalence of and risk factors for nonalcoholic fatty liver disease: the Dionysos nutrition and liver study. Hepatology.

[18] Organization WH (1998). Obesity: preventing and managing the global epidemic: report of a WHO consultation on obesity, Geneva, 3-5 June 1997.

[19] Paschos P, Paletas K (2009). Non alcoholic fatty liver disease and metabolic syndrome. Hippokratia.

[20] Hui E, Xu A, Bo Yang H, Lam KS (2013). Obesity as the common soil of non-alcoholic fatty liver disease and diabetes: Role of adipokines. J Diabetes Investig.

[21] Bril F, Lomonaco R, Cusi K (2012). The challenge of managing dyslipidemia in patients with nonalcoholic fatty liver disease. Clin Lipidol.

[22] Cusi K (2009). Role of insulin resistance and lipotoxicity in non-alcoholic steatohepatitis. Clin Liver Dis.

[23] Donati G, Stagni B, Piscaglia F, Venturoli N, Morselli-Labate A, Rasciti L (2004). Increased prevalence of fatty liver in arterial hypertensive patients with normal liver enzymes: role of insulin resistance. Gut.

[24] Wu S-J, Zou H, Zhu G-Q, Wang L-R, Zhang Q, Shi K-Q (2015). Increased levels of systolic blood pressure within the normal range are associated with significantly elevated risks of nonalcoholic fatty liver disease. Medicine.

[25] Michopoulos S, Chouzouri VI, Manios ED, Grapsa E, Antoniou Z, Papadimitriou CA (2016). Untreated newly diagnosed essential hypertension is associated with nonalcoholic fatty liver disease in a population of a hypertensive center. Clin Exp Gastroenterol.

